# Nonspecific Expression in Limited Excitatory Cell Populations in Interneuron-Targeting Cre-driver Lines Can Have Large Functional Effects

**DOI:** 10.3389/fncir.2020.00016

**Published:** 2020-04-27

**Authors:** Daniel Müller-Komorowska, Thoralf Opitz, Shehabeldin Elzoheiry, Michaela Schweizer, Eleonora Ambrad Giovannetti, Heinz Beck

**Affiliations:** ^1^Institute for Experimental Epileptology and Cognition Research, University of Bonn, Bonn, Germany; ^2^International Max Planck Research School for Brain and Behavior, University of Bonn, Bonn, Germany; ^3^Institute of Physiology and Pathophysiology, University Heidelberg, Bonn, Germany; ^4^Department of Electron Microscopy, Center of Molecular Neurobiology, University Medical Center Hamburg-Eppendorf, Hamburg, Germany; ^5^German Center for Neurodegenerative Diseases (DZNE) within the Helmholtz Association, Bonn, Germany

**Keywords:** Cre mouse line, cell-type specificity, optogenetics, interneuron, somatostatin, hippocampus, CA3

## Abstract

Transgenic Cre-recombinase expressing mouse lines are widely used to express fluorescent proteins and opto-/chemogenetic actuators, making them a cornerstone of modern neuroscience. The investigation of interneurons in particular has benefitted from the ability to genetically target specific cell types. However, the specificity of some Cre driver lines has been called into question. Here, we show that nonspecific expression in a subset of hippocampal neurons can have substantial nonspecific functional effects in a somatostatin-Cre (SST-Cre) mouse line. Nonspecific targeting of CA3 pyramidal cells caused large optogenetically evoked excitatory currents in remote brain regions. Similar, but less severe patterns of nonspecific expression were observed in a widely used SST-IRES-Cre line, when crossed with a reporter mouse line. Viral transduction on the other hand yielded more specific expression but still resulted in nonspecific expression in a minority of pyramidal layer cells. These results suggest that a careful analysis of specificity is mandatory before the use of Cre driver lines for opto- or chemogenetic manipulation approaches.

## Introduction

Transgenic Cre-recombinase expressing mouse lines are widely used in modern neuroscience to specifically direct the expression of fluorescent proteins or opto- and chemogenetic actuators to neuronal subtypes. Accordingly, they are a key element of most neuronal perturbation studies. Cre driver mouse lines have been extensively used to examine the function of interneuron subtypes *in vitro* and *in vivo*, with increasing numbers of Cre mouse lines for specific molecular markers of different interneuron subtypes (Taniguchi et al., 2011). Very commonly used are mice expressing Cre in subsets of GABAergic interneurons under the parvalbumin (PV) or somatostatin (SST) promoters. Those lines have allowed us to target two main categories of interneurons. In the hippocampus, PV^+^ cells include fast-spiking basket cells, axo-axonic cells, and interneuron types targeting proximal dendrites of pyramidal cells. SST^+^ cells, on the other hand, are regularly spiking and inhibit pyramidal cells at their distal dendrites (Lovett-Barron et al., 2012; Pelkey et al., 2017).

Commonly used Cre-lines have been widely assumed to be specific, with Cre-expression confined to the cells of interest. However, this assumption has been called into question in some cases. For example, in the widely used somatostatin–IRES-Cre line (SST-IRES-Cre, Taniguchi et al., 2011), a population of 5% of Cre-reporter positive cells were found to be fast-spiking PV^+^ cells (Hu et al., 2013). In the hippocampal CA1 subfield, this mouse line also targets a small (6%) population of fast-spiking interneurons as well as several putative pyramidal cells (Mikulovic et al., 2015). Opto- and chemogenetic studies in particular often depend on highly specific expression patterns to disseminate the function of neuronal subtypes. Even though these findings are worrisome, one defense of such mouse lines is that the absolute number of nonspecifically targeted cells is small. One could therefore assume that the observed *in vitro* and *in vivo* effects are dominated by the interneuron type in question.

Here we show that in SST-Cre mice (Savanthrapadian et al., 2014), recombination is not only induced in GABAergic interneuron types. Also, recombination occurs in a small subset of excitatory neurons largely confined to the CA3 pyramidal cell layer. Moreover, we find powerful functional effects of optogenetic activation that are not only contaminated by nonspecifically expressing glutamatergic cells but are completely lacking any interneuron contribution. Finally, we were also unable to find anatomical or functional differences between nonspecifically targeted cells and canonical CA3 pyramidal cells. This suggests that these cells are not a specific subtype of CA3 pyramidal cells. Further control experiments should be carried out in a region-specific manner, before using Cre-lines for the investigation of circuit function in behavior.

## Materials and Methods

### Transgenic Animals

All animal experiments were carried out according to the guidelines stated in Directive 2010/63/EU of the European Parliament on the protection of animals used for scientific purposes and were approved by authorities in Nordrhein-Westfalen (Landesamt für Natur, Umwelt und Verbraucherschutz Nordrhein Westfalen (LANUV), AZ 84-02.04.2014.A254).

The SST-Cre mouse line (C-SST^tm1Npa^) was kindly provided to us by Marlene Bartos and was described previously (Savanthrapadian et al., 2014). We hereafter refer to this line as the SST-Cre mouse line. In brief, the SST-Cre mice were generated by knocking NLS-Cre into the endogenous SST gene (Dinkel et al., 1999). The line was maintained by backcrossing with C57B6/N mice. Animals were bred heterozygously and were genotyped for Cre recombinase using the forward primer CCATCTGCCACCAGCCAG and the reverse primer TCGCCATCTTCCAGCAGG. Animals with an amplified fragment at 281 bp were classified as transgenic. For the cross-breeding experiments (Figure 6F), we used the Ai14 reporter line (Jackson Laboratories Stock No. 007914).

**Figure 1:**
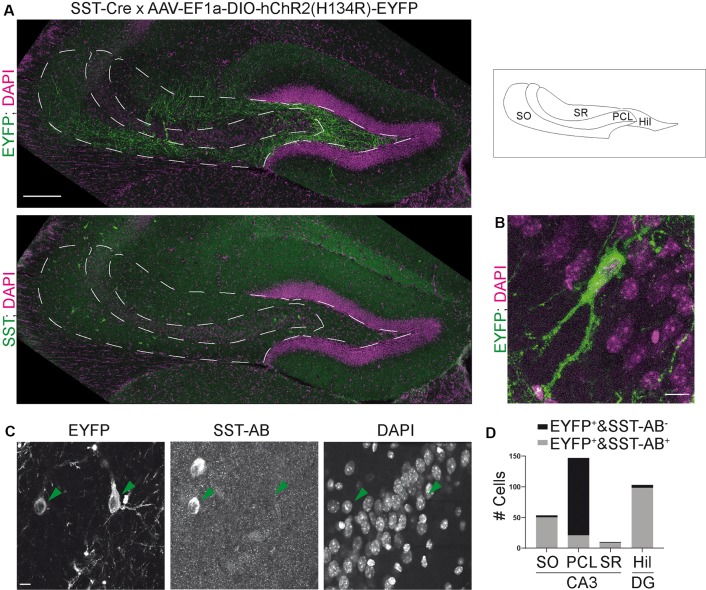
The somatostatin-Cre (SST)-Cre line is not specific for SST^+^ interneurons in CA3. **(A)** CA3 and the hilus of the dentate gyrus were virally transduced by intracranial stereotactic injection with a Cre dependent, enhanced yellow fluorescent protein (EYFP) expressing construct. Lower image shows SST staining in the same slice. 10× objective. Scale bar: 200 μm. Contrast adjusted for visualization. **(B)** EYFP^+^ cell in CA3 pyramidal cell layer (PCL) and two apical dendrites in stratum lucidum. Scale bar: 10 μm. **(C)** Example images showing two EYFP^+^ cells, one in stratum oriens (SO) and one in the PCL. The cell in SO is also SST positive, the cell in the PCL is SST negative. 40× magnification. Scale bar: 10 μm. **(D)** Quantification of SST colocalization from 40× images in six slices of five animals. Two of those were 300 μm thick acute slices.

**Figure 2:**
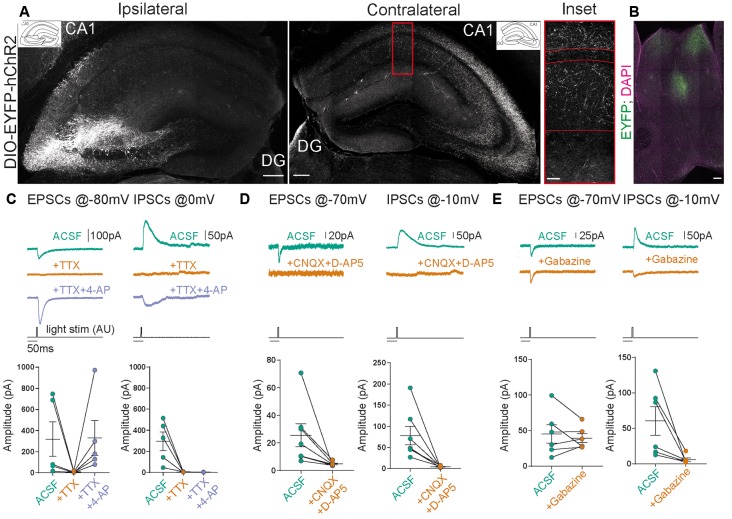
Stimulation of contralateral projections of CA3 neurons in the SST-Cre mouse line. **(A)** Confocal images from post-fixed acute slices of the ipsilateral injection site (left) and the contralateral hippocampus (right). The inset shows the fluorescent fiber signal in the contralateral hemisphere. Scale bars: 200 μm; inset: 20 μm. **(B)** Slice showing the projection from CA3 to the septum in the SST-Cre line. Scale bar: 100 μm. **(C–E)** Excitatory postsynaptic currents (EPSCs) and inhibitory postsynaptic currents (IPSCs) measured in contralateral CA1 pyramidal cells. Light stimulus is 5 ms long with 26 mW total light-fiber output. **(C)** The application of tetrodotoxin (TTX) alone abolished both excitatory and inhibitory currents. However, the co-application of TTX+4-AP recovered EPSCs but not IPSCs in all except one cell. Ratio *t*-test of dependent samples between artificial cerebrospinal fluid with sucrose (ACSF) and +TTX+4-AP one-tailed: EPSCs, *p* = 0.1412, *t* = 1.241; IPSCs, *p* < 0.0001, *t* = 13.18; *n* = 5 cells from three animals. **(D)** The application of CNQX+D-AP5 abolishes both EPSCs and IPSCs. Ratio *t*-test of dependent samples one-tailed: EPSCs, *p* = 0.0017, *t* = 4.681; IPSCs, *p* < 0.0001, *t* = 8.082; *n* = 7 cells from four animals. **(E)** The application of Gabazine does not affect EPSCs but inhibits IPSCs. Ratio *t*-test of dependent samples one-tailed: EPSCs, *p* = 0.4818, *t* = 0.04799; IPSCs, *p* = 0.0021, *t* = 4.947; *n* = 6 cells from three animals. All responses were recorded at 26 mW fiber output.

**Figure 3:**
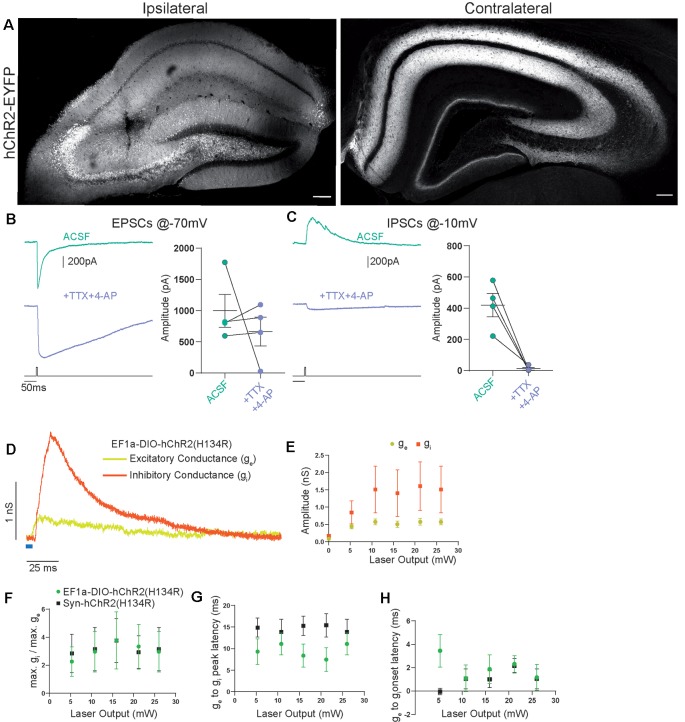
Contralateral projections originating from Cre-expressing cells in CA3 vs. the general CA3 neuron population are functionally indistinguishable. **(A)** Confocal images from post-fixed acute slices of the ipsilateral injection site (left) and the contralateral hippocampus (right). Unconditional viral expression. **(B)** EPSCs and **(C)** IPSCs (right) before and after bath application of TTX and 4-AP measured in contralateral CA1 pyramidal cells. 5 ms light stimulation at 26 mW fiber output. Ratio *t*-test of dependent samples one-tailed: EPSCs, *p* = 0.2284, *t* = 0.8519; IPSCs, *p* = 0.0069, *t* = 5.200, *n* = 4 cells from two animals. **(D)** Example from conductance analysis of fibers in the SST-Cre mouse line, conditionally expressing. Twenty-six microwatt light fiber output and 5 ms light stimulation. Excitatory conductance was calculated from gabazine traces. Inhibitory conductance was calculated from gabazine subtracted traces. **(E)** Quantification of excitatory and inhibitory peak conductance at different laser powers. 2-way ANOVA Greenhouse-Geisser corrected: main effects, Laser Output: *p* = 0.0422, *DF* = 5, *F*_(1.182,7.091)_ = 5.849, Conductance Type: *p* = 0.2189, *DF* = 1, *F*_(1.000,6.000)_ = 1.885. Interaction: *p* = 0.2527, *DF* = 5, *F*_(1.115,6.693)_ = 1.600. *n* = 6 cells from three animals, same as EF1a-DIO-hChR2(H134R) in **(F–H)**. **(F)** Quantification of conductance ratios (inhibitory peak conductance divided by excitatory peak conductance) for conditional viral expression (EF1a-DIO-hChR2(H134R)) and unconditional expression (Syn-hChR2(H134R)). 2-way ANOVA Greenhouse-Geisser corrected: main effects, Laser Output: *p* = 0.1406, *DF* = 4, *F*_(1.393,15.33)_ = 2.341, Expression Type: *p* = 0.9614, *DF* = 1, *F*_(1,11)_ = 0.002455. Interaction: *p* = 0.7974, *DF* = 4, *F*_(4,44)_ = 0.4143. **(G)** Quantification of latency between excitatory peak conductance and inhibitory peak conductance. 2-way ANOVA Greenhouse-Geisser corrected: main effects: Laser Output: *p* = 0.6446, *DF* = 4, *F*_(1.720,18.92)_ = 0.4014, Expression Type: *p* = 0.1766, *DF* = 1, *F*_(1,11)_ = 2.085. Interaction: *p* = 0.0320, *DF* = 4, *F*_(4,44)_ = 2.912. **(H)** Quantification of latency between excitatory conductance onset and inhibitory conductance onset. 2-way ANOVA Greenhouse-Geisser corrected: main effects, Laser Output: *p* = 0.6474, *DF* = 4, *F*_(2.306,25.37)_ = 0.4853, Expression Type: *p* = 0.1759, *DF* = 1, *F*_(1,11)_ = 2.092. Interaction: *p* = 0.3588, *DF* = 4, *F*_(4,44)_ = 1.121. EF1a-DIO-hChR2(H134R) *n* = 6 cells from three animals; Syn-hChR2(H134R) *n* = 7 cells from three animals.

**Figure 4:**
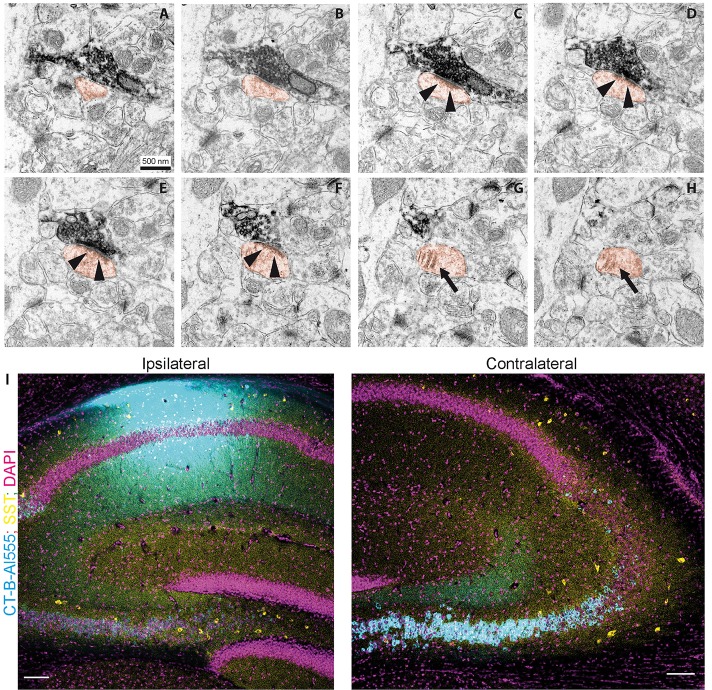
Contralaterally projecting axons originating from Cre-expressing neurons in CA3 are excitatory. **(A–H)** miniSOG positive electron-dense structure making presynaptic contact on a spine (orange) in CA1 SO. Arrows in **(C–F)** show postsynaptic density. Arrows in **(G–H)** show the spine apparatus. Scale bar: 500 nm. **(I)** Cholera toxin-B tracing in CA1. Ipsilateral injection of CT-B subunit in CA1. Contralateral, retrogradely traced cells (cyan) and SST immunoreactive cells (yellow). Scale bar: 200 μm.

**Figure 5:**
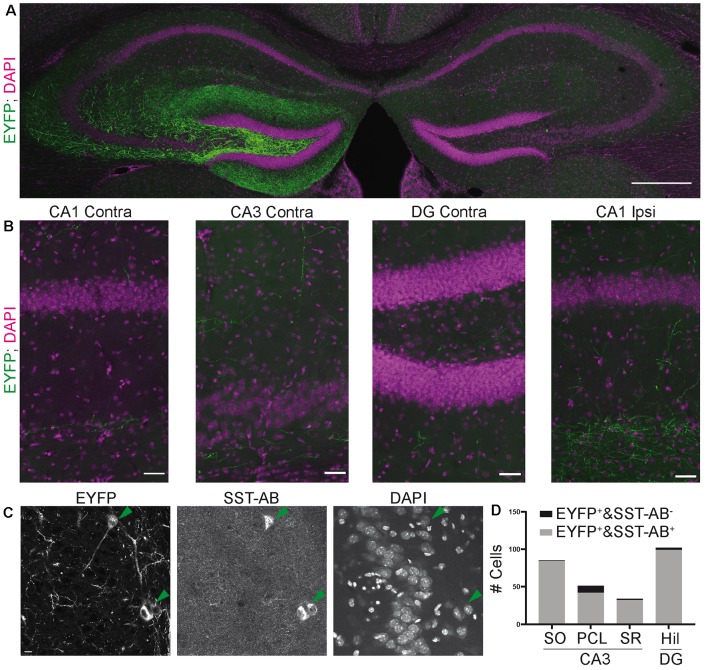
Viral transduction in the SST-IRES-Cre line results in a more specific expression. **(A)** Overview image of the ipsilateral injection site (left) and contralateral hemisphere. Virtually no contralateral fibers were observed (compare with Figure 2A). Mosaic merge, 20× magnification. Scale bar: 200 μm. **(B)** Enlarged views from **(A)** showing the lack of contralateral fiber signal. Even in ipsilateral CA1, fiber signal is constrained to stratum lacunosum moleculare, as would be expected from OLM-interneuron specific labeling. Scale bar: 20 μm. **(C)** Image of EYFP positive cells in CA3 SO. 40× magnification confocal microscope. Scale bar: 10 μm. **(D)** Quantification of EYFP and SST positive cells, 15 slices from six SST-IRES-Cre animals.

**Figure 6:**
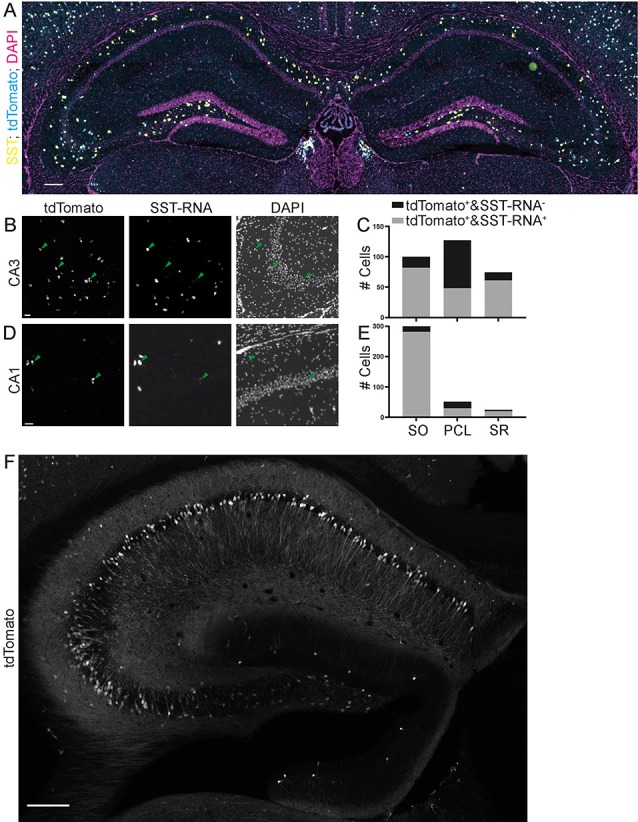
Specificity of expression in SST-IRES-Cre mice achieved by crossing with a reporter mouse line. Images **(A,B,D)** from the Allen Brain Institute. The SST-IRES-Cre mouse line was crossed with the tdTomato reporter line Ai14. **(A)** Experiment 167643437, image ID 167643516. Contrast auto-adjusted and lookup tables changed. Scale bar: 100 μm. **(B–E)** Example images cropped from **(A)**, contrast unadjusted. Quantification on the right. Scale bar: 20 μm. **(F)** The SST-Cre mouse line crossed with the Ai14 reporter line. Scale bar: 200 μm.

B6N.Cg-*Sst^tm2.1(cre)Zjh^*/J mice (SST-IRES-Cre, stock number 018973, The Jackson Laboratory) express Cre recombinase (IRES-Cre-pA cassette) in the 3′UTR of the Somatostatin locus (Taniguchi et al., 2011). We consistently refer to this line as the SST-IRES-Cre mouse line. These mice were crossbred to B6.Cg-Tg(APPswe, PSEN1dE9) 85Dbo/Mmjax (Jankowsky et al., 2004) mice and only Cre heterozygous offspring were used for experiments. Mice used in this study were negative for the APP/PS1 transgene. The wild type C57BL/6J animals were negative for both the APP/PS1 gene and SST-Cre. Mice were genotyped for SST-IRES-Cre with the following primers: GGGCCAGGAGTTAAGGAAGA; TCTGAAAGACTTGCGTTTGG and TGGTTTGTCCAAAC-TCATCAA. We genotyped for the APP/PS1 gene using AATAGAGAACGGCAGGAGCA; GCCATG-AGGGCACTAATCAT; CTAGGCCACAGAATTGAAAGATCT; GTAGGTGGAA-ATTCTAGCATCATCCW.

### Stereotaxic Intracranial Viral Injections

Animals were anesthetized with a ketamine/rompun or a fentanyl/midazolam/medetomidine mixture i.p. Animals also received ketoprofen analgesia (5 mg/kg, 0.1 ml/10 g body weight) before the surgery and daily 2 days after the surgery. Viral particles (250 nl at a rate of 100 nl/min) were injected into CA3/hilus of the right hemisphere at the following coordinates relative to Bregma: 2.3 mm posterior; 1.6 mm lateral (1.75 for SST-IRES-Cre animals); 2.5 mm ventral. We used rAAV1/2-Ef1a-DIO-hChR2(H134R)-EYFP-WPRE-pA (received as a gift from Karl Deisseroth, Addgene plasmid # 20298; http://n2t.net/addgene:20298; RRID:Addgene_20298) for Cre-mediated opsin expression, AAV1/2-Ef1a-DIO-Syp-miniSOG-t2A-mCherry-WPRE-hPa (received as a gift from Roger Tsien; Shu et al., 2011) for electron microscopy experiments and AAV1/2.Syn-hChR2(H134R)-EYFP (received as a gift from Karl Deisseroth, Addgene plasmid # 26973; http://n2t.net/addgene:26973; RRID:Addgene_26973) for general expression. Cholera Toxin subunit B (CT-B, 50 nl), Alexa Fluor 555 conjugate (C-34775, Thermo Fischer) was injected into CA1 at Bregma coordinates: 1.9 mm posterior; 1.5 mm lateral; 1.7 ventral. Mice were used for electrophysiological experiments 4–5 weeks after viral injection.

### Somatostatin Immunostaining and Colocalization Analysis

Animals were transcardially perfused with 4% PFA and the brains were post-fixed with 4% PFA overnight at 4°C. The brains were washed in PBS the next day and slices of the dorsal hippocampus were cut on a vibratome (HM 650V; Thermo Scientific) at 50 μm. Acute 300 μm slices were postfixed for 1 h in 4% PFA. After washing, slices were left in a blocking solution, consisting of 3% BSA in 0.25% PBS-T, for 2 h at room temperature (RT). Then the primary antibody, rabbit anti-SST (T-4102, Peninsula Laboratories International), was applied 1:500 in blocking solution overnight shaking at 4°C. The following day slices were left at RT for 30 min and washed in a blocking solution. The secondary antibody, donkey anti-rabbit IgG, Alexa fluor 647 (ab150075, Abcam), was applied 1:500 overnight shaking at 4°C. Finally, slices were washed, stained with 1:1,000 DAPI for 30 min at RT shaking and mounted with aqua-poly mount. The SST staining for the cholera toxin-B (CT-B) injected animals followed a slightly different protocol where slices were blocked with 5% donkey serum instead of BSA and the secondary antibody was donkey anti-rabbit IgG FITC 1:500 (ab6798, Abcam).

For colocalization, 40× confocal images were taken with a Leica SP8 confocal microscope. Enhanced yellow fluorescent protein (EYFP) positive cells were sought out in dentate gyrus and CA3. Colocalization was quantified manually by inspecting signals in the SST channel at the somatic localization of the EYFP signal. Figure 1C shows representative examples for both SST colocalizing and non-colocalizing cells. Automatic quantification was not feasible because the eYFP neuropil signal did not allow automatic soma segmentation. Overview image (Figures 5A,B) was taken on a spinning-disk microscope.

### *In vitro* Electrophysiology

Adult mice were anesthetized with isofluorane, rapidly decapitated and the dissected brains were transferred to ice-cold, carbogenated artificial cerebrospinal fluid with sucrose (ACSF; in mM: NaCl, 60; sucrose, 100; KCL, 2.5; NaH_2_PO_4_, 1.25; NaHCO_3_, 26; CaCl_2_, 1; MgCl_2_, 5; glucose, 20; from Sigma-Aldrich) and sliced to 300 μm. Slices were then transferred to ACSF at 37°C and left for 20 min. They were then transferred to carbogenated ACSF without sucrose (NaCl, 125; KCL, 3.5; NaH_2_PO_4_, 1.25, NaHCO_3_, 26; CaCl_2_, 2; MgCl_2_, 2; glucose, 20; from Sigma-Aldrich) and were used for experiments after at least 1 h at RT. All experiments were performed in the same ACSF without sucrose at RT. The intracellular solution for voltage-clamp experiments contained in mM: Cs methanesulfonate, 120; MgCl_2_, 0.5; 2-(4-(2-Hydroxyethyl)-1-piperazinyl)-ethansulfonsäure (HEPES), 5; Ethylenglycol-bis(aminoethyl ether)-N, N,N′,N′-tetraessigsäure (EGTA), 5; Adenosine 5′-triphosphate disodium salt (Na_2_-ATP), 5; N-(2,6-Dimethylphenylcarbamoylmethyl)triethylammonium chloride (QX 314 Cl^−^), 5; from Sigma Aldrich. For pharmacology, we furthermore used 10 μM gabazine (SR 95531 hydrobromide; Tocris), 1 μM tetrodotoxin (TTX, Tocris), 200 μM 4-aminopyridine (4-AP, Sigma Aldrich), 50 μM 6-Cyano-7-nitroquinoxaline-2,3-dione disodium salt (CNQX, Tocris), 200 μM D-(-)-2-Amino-5-phosphonopentanoic acid (D-AP5, Tocris). All these compounds were applied in the recording chamber for at least 10 min before continuing measurements. Most were applied for 20 min.

Patch-clamp experiments were performed with an Axopatch 200B and digitized on a Digidata 1322A or Digidata 1550B plus HumSilencer (Molecular Devices). Light stimulation was performed with an Omicron Luxx 473 nm laser attached to a light fiber submerged in the ACSF. Light stimuli were 5 ms long unless otherwise stated.

For the conductance analysis, we assumed a chloride reversal potential of −80 mV (−78.9 mV calculated with Nernst equation) and a cation reversal potential of 0 mV. The excitatory conductance was calculated from a current trace measured at a holding voltage near the chloride reversal with gabazine washed-in, to ensure pure excitatory response. To isolate the inhibitory conductance, we subtracted the pure excitatory response at a depolarized holding voltage from the mixed response in normal ACSF.

In Figure 2C we only included cells that showed complete block by TTX wash-in. We excluded one cell that did not show a complete block, which is likely due to a wash-in failure.

### Electron Microscopy With miniSOG Photooxidation

SST-Cre animals were virally transduced with AAV1/2-Ef1a-DIO-Syp-miniSOG-t2A-mCherry-WPRE-hpA. Three weeks later, mice were transcardially perfused with Ringer solution followed by 4% formaldehyde in 0.15 M cacodylate-buffer. Brains were removed and post-fixed overnight at 4°C. Coronal slices (100 μm) were taken on a vibratome and slices with distinct mCherry fluorescence were chosen. Slices were fixed with 2% glutaraldehyde for 30 min, washed with ice-cold cacodylate-buffer, and blocked for 20 min in a solution containing 20 mM glycine, 10 mM KCN, and 20 mM aminotriazoline in cacodylate-buffer. For photooxidation, slices were immersed in freshly prepared and filtered (0.22 μm) 3,3’-diaminobenzidine (DAB) solution (1 mg/ml DAB in 0.1 M cacodylate-Buffer, pH 7.4) that was aerated with oxygen. The miniSOG was activated with a blue light (FITC filter set: EX470/40, DM510, BA520) applied through a LUMPlanFl 60 × NA 0.90 W at an inverted Olympus microscope equipped with a 100 W HBO-Lamp. Light was applied for 20 min and fresh DAB solution was exchanged after 10 min. After illumination, slices were stored in cacodylate-buffer for further processing.

After photoconversion, the converted region containing DAB reaction product in the hippocampus was documented and images were taken at a Zeiss Axiophot light microscope. Thereafter the sections were rinsed three times in 0.1 M sodium cacodylate buffer (pH 7.2–7.4; Sigma-Aldrich, Germany) and incubated with 1% osmium tetroxide (Science Services, Germany) in cacodylate buffer for 20 min on ice. The osmication of sections was followed by dehydration through ascending ethyl alcohol concentration steps and rinsing twice in propylene oxide (Carl Roth, Germany). Infiltration of the embedding medium was performed by immersing the sections first in a mixture of 2:1 of propylene oxide and Epon (Carl Roth, Germany) then in a 1:1 mixture and finally in neat Epon and polymerized at 60°C for 48 h. The region of interest was dissected and ultrathin sections (60 nm) were prepared with a Leica Ultracut UC7. Images were taken using an EM902 transmission electron microscope (Zeiss, Germany) equipped with a CCD in lens 2K digital camera and running the ImageSP software (Tröndle, Moorenweis, Germany).

### Quantification and Statistical Analysis

We used Python with Matplotlib (Hunter, 2007) and GraphPad Prism for plotting. Electrophysiological data were analyzed manually in Clampfit (Molecular Devices) or with python and NumPy (van der Walt et al., 2011). To load .abf files into python we used the python-neo package (Garcia et al., 2014). GraphPad Prism was used for statistical analysis. We used the *t*-test to compare 2 groups and two-way ANOVA to compare two groups across multiple conditions.

For the quantification of the Allen Brain Institute data (Oh et al., 2014), we used the Allen Software Development Kit to download .jpg images. tdTomato positive cells were segmented by maximum entropy thresholding, erosion, dilation and the particle counter in ImageJ (Schindelin et al., 2012). Colocalization with fluorescent *in situ* hybridization probe was assessed manually. In total, we quantified 23 images of the dorsal hippocampus from four experiments (Table 1). A detailed technical description can be found in the Transgenic Characterization whitepaper: http://help.brain-map.org/display/mouseconnectivity/Documentation.

**Table 1:** Experiments and images from the Allen Brain Institute used for the quantification in Figures 6, 7.

Line	Experiment	Img ID					
SST-IRES-Cre	182530118	182530130	182530134	182530136	182530140	182530142	182530156
	167643437	167643500	167643502	167643504	167643514	167643516
Pvalb-IRES-Cre	81657984	81636703	81636705	81636709	81636711	81636713	81636715
	111192541	111192610	111192612	111192625	111192627	111192629	

*All images can be found here: http://connectivity.brain-map.org/transgenic*.

## Results

### The SST-Cre Line Is Not Specific for SST^+^ Interneurons in CA3

Somatostatin (SST) positive interneurons in CA3 are located predominantly in stratum oriens (SO) and stratum radiatum (SR). SST positive cells have a characteristic dendrite morphology, with most of the dendritic arbor confined to the same sublayer as the soma (Freund and Buzsáki, 1996). We expressed a construct that leads to Cre-dependent expression of EYFP in the CA3 region of heterozygous SST-Cre mice using rAAV-dependent gene transfer. We found EYFP expression in cells of the pyramidal cell layer (PCL; Figure 1A). In SO and SR, cells also expressed EYFP but the signal there was almost dominated by the neuropil. EYFP^+^ cells in the PCL showed features typical for CA3 pyramidal cells (Figure 1B) such as thorny excrescences on apical dendrites.

To determine if these EYFP^+^ cells are also SST^+^, we immunostained for SST. This revealed that EYFP expression was highly specific for SST^+^ interneurons in SO, where 50/53 EYFP^+^ cells expressed SST. Similarly, in SR 9/10 EYFP^+^ cells expressed SST. In marked contrast, we found that a minority of EYFP^+^ cells in the pyramidal cell layer of CA3 coexpressed SST (21/147 cells; Figures 1C,D data from six slices of five animals). Injection of the Cre-dependent virus into control animals lacking Cre-recombinase activity did not lead to EYFP expression (nine slices, three animals).

These results show that Cre recombinase is not only targeted to SST^+^ interneurons in the adult hippocampus. It is also expressed in pyramidal-like neurons within the PCL that is devoid of detectable somatostatin levels, leading to the targeting of these cells even with viral gene transfer in adult animals. In contrast, the SST-Cre mouse line showed local specificity in CA3 SO, SR and the hilus of the dentate gyrus.

### Commissural Projections Make Direct Excitatory Connections in Contralateral CA1

Does a relatively small number of CA3 neurons targeted in SST-Cre mice have a measurable functional impact on neuronal networks? CA3 pyramidal neurons are known to make extensive long-range connections to the contralateral hippocampus (Buzsáki and Czéh, 1981; Buzsáki and Eidelberg, 1982; Finnerty and Jefferys, 1993) and the septum (Risold and Swanson, 1997). We therefore examined if the small number of CA3 neurons targeted in SST-Cre mice is sufficient to generate detectable contralateral projections. Unilateral rAAV injection in the CA3 region of SST-Cre mice led to a strong axonal EYFP signal in the contralateral hippocampus (Figure 2A) and the septum (Figure 2B). The axon distribution was as described for CA3 pyramidal cells, with EYFP-expressing axons mainly in SO and SR of both the CA1 and CA3 regions.

Contralateral projections have been described not only for CA3 pyramidal neurons but also for inhibitory hippocampal interneurons including SST-expressing subtypes (Zappone and Sloviter, 2001; Eyre and Bartos, 2019). We, therefore, went on to further characterize the functional properties of contralaterally projecting axons, to assess: (i) if they correspond to excitatory projections arising from CA3 pyramidal neurons; and (ii) if they are sufficiently numerous to cause significant physiological effects. To this end, we obtained patch-clamp recordings from CA1 pyramidal neurons in mice expressing hChR2 in the contralateral CA3 region in SST-Cre mice. This allowed us to perform light-based stimulation of contralaterally projecting axons while recording from CA1 pyramidal neurons. To separate excitatory from inhibitory neurotransmission, we voltage-clamped CA1 neurons to different holding voltages. Currents at −80 or −70 mV were evoked close to the chloride reversal potential and are therefore dominated by excitatory postsynaptic currents (EPSCs), whereas currents evoked at 0 or −10 mV are dominated by inhibitory postsynaptic currents (IPSCs). In all CA1 pyramidal neurons, blue light illumination reliably evoked both excitatory and inhibitory currents (Figures 2C–E). To ascertain which of these components are monosynaptic, we applied the Na^+^ channel blocker tetrodotoxin (TTX, 1 μM), which invariably blocked synaptic transmission completely. Coapplying TTX with 4-aminopyridine (4-AP, 200 μM) enables direct light-based transmitter release from terminals expressing ChR2, and thus indicates monosynaptic connections. Coapplication of 4-AP recovered EPSCs, but not IPSCs (Figure 2C; EPSCs 217%, IPSCs 1% of baseline). The recovery of EPSCs but not IPSCs indicates that contralateral projections in SST-Cre mice are excitatory. Additionally, these results indicate that the light-evoked IPSCs are due to polysynaptic recruitment of interneurons. This idea is supported by the temporal delay between excitatory and inhibitory conductances (Figures 3G,H). Consistent with polysynaptic recruitment of inhibitory interneurons, light-evoked IPSCs were abrogated by blocking glutamatergic transmission with CNQX (50 μM) and D-AP5 (200 μM; Figure 2D; EPSCs 29%, IPSCs 8% of baseline). Finally, we show that—as expected—light-evoked IPSCs were sensitive to the GABA-A blocker gabazine (10 μM; Figure 2E; EPSCs 114%, IPSCs 14% of baseline).

Taken together, we found no evidence for direct commissural inhibition from SST^+^ interneurons from CA3 to CA1. Instead, direct excitatory transmission recruited strong polysynaptic inhibition.

### Properties of Commissural Axons Targeted Unconditionally or in an SST-Cre Mouse Line Are Functionally Indistinguishable

To investigate if this is consistent with the canonical CA3 to CA1 commissural projection, we induced broad expression of ChR2 in all CA3 cell types using viral gene transfer of an unconditional construct leading to expression of EYFP-hChR2. Light-based manipulations should be dominated by the activity of pyramidal cells, since they vastly outnumber other neuronal subtypes. Virus injection resulted in a strong fluorescence signal in CA1, CA3, and DG that was dominated by fiber signal at the injection site (Figure 3A). Contralateral to the injection site, we found prominent labeling of axons in CA1 and CA3 in both SR and SO as well as the inner molecular layer of the DG. The DG fiber pattern was consistent with the commissural mossy cell projection and the fiber patterns in CA1 and CA3 with the commissural CA3 projection. We again assessed the monosynaptic transmission onto contralateral CA1 pyramidal cells using the combined application of TTX and 4-AP (1 μM, 200 μM) and found that it completely inhibited IPSCs (Figures 3B,C; EPSCs 88%, IPSCs 4% of baseline). Next, we asked if there are quantitative differences between the SST-Cre fibers and the unconditionally transduced fibers. We converted the pharmacologically isolated currents (Figure 2E) to conductances (Figure 3D) according to holding and reversal potentials (see “Materials and Methods” section). Because the density of EYFP-hChR2 positive fibers is much larger in the unconditional case, the absolute conductances cannot be compared meaningfully. However, because the inhibition is polysynaptic, it is expected to scale to some extent with the excitation. Therefore, the ratio between excitation and inhibition can give insights into differential recruitment in the micro-network.

We found that in the SST-Cre line, the inhibitory conductance was stronger than the excitatory one (Figures 3D,E). Comparing the SST-Cre line with the unconditional case, we did not detect a difference between the ratios of maximum inhibition and excitation (Figure 3F). In both cases, the amplitude of inhibition was larger than that of inhibition for different strengths of light-based stimulation. Furthermore, the latencies between the onset of excitation and inhibition showed no significant difference (Figure 3H) and were consistent with values found in CA3 to CA1 Schaffer collateral projections (Pouille and Scanziani, 2001). However, the latencies between the peak of the excitatory conductance and the inhibitory conductance showed a significant interaction between laser output and the type of expression. The main effects were not significant (Figure 3G, Greenhouse-Geisser corrected 2-way ANOVA).

### Commissural CA3 Fibers Make Synaptic Contacts on Spines and Originate Primarily From PCL Cells

To further confirm that contralateral projections are excitatory, we used miniSOG photooxidation to generate electron-dense labeling in contralateral CA1 SO localized to fibers with Cre recombinase activity in the SST-Cre line (Figure 4). Of 70 miniSOG positive structures, 40 were presynaptic boutons making postsynaptic contacts. All 40 structures made contact on a spine, four of them made contact on two spines. Serial imaging sections of 25 boutons showed that 22 of them unambiguously made contact on spines (Figures 4A–H, quantification from two slices). The other three boutons were not entirely sectioned. The types of most synaptic contacts could not be defined clearly because of the electron-dense labeling in the pre-synapse. However, the postsynaptic densities that are clearly in the imaging plane appear asymmetric. Together with the fact that they all contact spines, this data suggests that the direct contacts are predominantly excitatory, and we found no evidence for direct inhibitory contacts in CA1 SO.

Next, we used retrograde tracing in CA1 with CT-B to determine which cell types project to contralateral CA1 (Figure 4I). We found that virtually all projecting cells were in the CA3 pyramidal cell layer. With the SST staining we identified 81 cells (12 slices from four animals), none of which was CT-B positive. This data suggests that somatostatin interneurons are not part of the commissural projection.

Finally, we related our findings to the more commonly used SST-IRES-Cre mouse line (Taniguchi et al., 2011). For this purpose, we used data from the Allen Brain Institute. We also virally injected SST-IRES-Cre animals for direct comparison of genetic and viral expression.

### Viral Gene Transfer Leads to More Specific Expression Compared to the Use of Reporter Mouse Lines

Do these findings generalize to other, commonly used SST-Cre mouse lines? The SST-IRES-Cre line (Taniguchi et al., 2011) has been widely used, with 203 publications relating to it according to Jackson Laboratories (as of 11.10.2019). We therefore examined if viral transduction in adult SST-IRES-Cre animals also leads to nonspecific expression in non-SST expressing neurons. We found that viral gene transfer in SST-IRES-Cre mice led to a much more specific expression pattern in CA3 compared to the SST-Cre line. Labeling of contralaterally projecting axons was almost completely absent in the contralateral CA1 region. Few axons were present in the contralateral CA3 region and DG, potentially corresponding to DG interneuronal axons (Figures 5A,B). The fiber signal in the ipsilateral CA1 region was strongest in stratum lacunosum moleculare, as would be expected for SST positive oriens lacunosum moleculare cells. Somatostatin staining confirmed that viral expression is highly specific for somatostatin positive cells (Figure 5C). Quantification in 15 slices from 6 animals showed that 257/272 (94.5%) EYFP^+^ cells were also SST positive. In the CA3 SO 84/85 (98.8%), in SR 32/34 (94.1%) and DG 99/102 (97.1%) of EYFP^+^ cells were SST positive. In the CA3 PCL, the specificity was somewhat lower (42/51 cells, 82.4%), Thus, also in the SST-IRES-Cre mouse, specificity was least in the PCL of CA3, with almost 20% of neurons lacking SST expression. However, SST-IRES-Cre mice are more selective than SST-Cre mice following viral transduction.

Since many experimenters also breed Cre driver mouse lines with conditional mouse lines expressing fluorescent proteins or opto- or chemogenetic actuators, we also evaluated the specificity of both the SST-Cre or the SST-IRES-Cre line when they were crossed with the Ai14 tdTomato reporter mouse line. For the SST-IRES-Cre line, we used the Allen Brain Institute transgenic characterization data of the mouse connectome project (Oh et al., 2014). We used two experiments in which the SST-IRES-Cre mouse line was crossed with the Ai14 tdTomato reporter mouse line and fluorescent *in situ* hybridization (FISH) was performed for SST. We found that in these experiments, the SST-IRES-Cre mouse line is nonspecific in CA3, with only 48/127 (37.8%) tdTomato^+^ cells being SST-mRNA^+^ in the PCL, 82/100 (82%) in SO and 61/74 (82.4%) in SR (Figures 6A–C). The CA1 area also contained some SST- cells in the PCL but appeared overall more specific with 29/51 (56.9%) tdTomato^+^ cells being SST-mRNA positive, 281/299 (94%) in SO and 20/24 (83.3%) in SR (Figures 6A,D,E). Thus, using breeding with reporter mouse lines, even the more specific SST-IRES-Cre mouse line lacks sufficient specificity, in particular in the subfield most affected in the SST-Cre mouse line.

We also crossed SST-Cre mice with Ai14 tdTomato reporter mice. This approach led to an even more unselective pattern of expression, with pyramidal-like tdTomato expressing cells in the PCL of CA3, but also the CA2 and CA1 subregions. We also found a very small number of granule cell-like neurons in the granule cell layer of the dentate gyrus (Figure 6F) that were not observed in virally transduced animals. Since this pattern was unselective, it was not further quantified.

**Figure 7:**
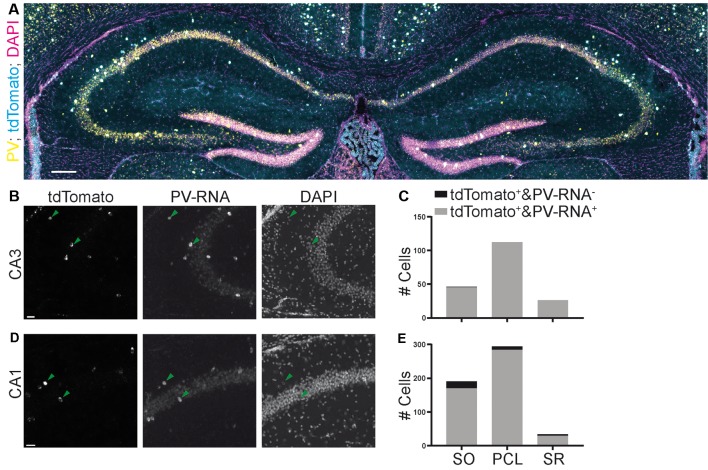
Specificity of expression in a parvalbumin (PV)-Cre mouse line achieved by crossing with a reporter mouse line images **(A,B,D)** from the Allen Brain Institute. **(A)** Experiment 111192541, image ID 111192610. Contrast auto-adjusted and lookup tables changed. **(B–E)** Example images cropped from **(A)** contrast unadjusted. Quantification on the right. Scale bars: 100 and 20 μm.

Finally, we also assessed the quality of a third commonly used mouse line targeting PV containing interneurons. We quantified the colocalization of Cre-induced recombination with PV expression in the Pvalb-IRES-Cre mouse line (Hippenmeyer et al., 2005). We found that this mouse line was much more specific than both SST-Cre mouse lines in both the CA3 and CA1 regions (Figures 7A–E; CA3: 45/46, 97.8% SO; 112/112, 100% PCL; 26/26, 100% SR. CA1: 170/191, 89% SO; 284/294, 96.6% PCL; 29/34, 85.3% SR).

## Discussion

We show that CA3 PCs that are nonspecifically targeted in an SST-Cre mouse line (Savanthrapadian et al., 2014) make functional connections indistinguishable from those of canonical CA3 PCs. While the specificity of SST-Cre lines has been questioned before, the functional relevance of the nonspecific expression of Cre-recombinase was unknown. Estimating the potential effects of nonspecific expression is essential for neuronal perturbation studies that seek to isolate the function of specific cell-types. Our data suggest that studies that perturb SST cells in CA3 with the SST-Cre line would be massively confounded by Cre-recombinase expression in CA3 pyramidal cells. We also demonstrate that a commonly used SST-IRES-Cre line is more specific, but still exhibits low levels of nonspecific Cre expression in particular in specific subfields, in this case, the Ca3 pyramidal cell layer. We also show that crossing SST-Cre and SST-IRES-Cre mouse lines with a reporter mouse line leads to more extensive nonspecific expression compared to viral gene transfer. This may be due to the widespread activity of the SST promoter in non-SST interneurons during early development (Zingg et al., 1984; Lowe et al., 1987; Xiang et al., 2001). Additionally, the activity of the SST promoter may be regulated by neuronal activity (Gonzalez and Montminy, 1989).

An additional comparison is of interest: nonspecific Cre expression was more widespread in the SST-Cre compared to the SST-IRES-Cre mouse line. This was surprising as both Cre mouse lines were generated using a knockin strategy into the endogenous SST gene. However, the targeting strategy was different. While the SST-IRES-Cre mouse was generated by inserting an IRES-Cre cassette immediately after the STOP codon (Taniguchi et al., 2011), the SST-Cre mouse was generated by knocking NLS-Cre into the endogenous SST gene (Savanthrapadian et al., 2014). It is thus possible that these different targeting strategies, with a different relationship of the inserted gene sequence to the endogenous SST promoter, affect the expression pattern of Cre recombinase.

### How Relevant Are These Findings for Other Cre Mouse Lines?

We demonstrate wide-spread physiological effects of nonspecific Cre-expression in the SST-Cre mouse line but have found anatomical evidence for a less pronounced nonspecific genetic expression in the SST-IRES-Cre mouse line. Indeed, specificity issues with an SST-IRES-Cre mouse line were raised previously (Taniguchi et al., 2011). Moreover, a further study has found targeting of a large number (31%) of slow-spiking cells in the CA1 PCL, also consistent with nonspecific genetic expression (Mikulovic et al., 2015). Specificity can vary widely between Cre lines and brain areas, as our comparison of the SST-IRES-Cre and the Pvalb-IRES-Cre lines shows. Therefore, specificity should not be generalized lightly to other Cre mouse lines or even to other brain areas in the same mouse line. We suggest that pending careful quantitative analysis in all the subregions under investigation in the specific study, caution is warranted in assuming specificity.

### Do SST-Expressing Interneurons Make Contralateral Connections?

In addition to CA3 pyramidal cells, the SST-Cre mouse line targets SST^+^ INs in CA3. We found that the projection of the contralateral CA1 region arises mainly from nonspecifically targeted pyramidal cells. We found no evidence for direct inhibition from SST^+^ interneurons onto contralateral CA1 PCs in our patch-clamp experiments. Even slices with nonconditional ChR2 expression did not exhibit monosynaptic inhibition, despite all inhibitory cell types being targeted. Furthermore, our anatomical EM data showed no evidence for inhibitory synapses in contralateral CA1 SO. Finally, the CT-B data did not reveal cells outside CA3 PCL projecting to contralateral CA1. This leads us to the conclusion that an inhibitory CA3 to contralateral CA1 connection is extremely weak or nonexistent and SST^+^ interneurons do not contribute to it.

Although we focused on the CA3 and CA1 subfields, we noted a very sparse fiber signal in the outer molecular layer of DG in the SST-Cre line. This is in line with previous anatomical evidence showing a commissural projection with a GABAergic component (Deller et al., 1995; Zappone and Sloviter, 2001). However, using *in vivo* patch-clamp and optogenetics we did not find evidence for a functional connection onto granule cells (data not shown).

Eyre and Bartos (2019) have also assessed interhemispheric connections of inhibitory interneurons using unilateral viral gene transfer in either GAD2-Cre and the SST-IRES-Cre mouse lines. In the SST-IRES-Cre mouse line, virus injection into the CA3 regions revealed a large number of cells in the CA3 PCL far exceeding cell numbers in CA3 stratum radiatum or oriens (see Eyre and Bartos, 2019; Figure 2B). This distribution of targeted cells in CA3 is reminiscent of the SST-Cre mouse line described in this article (Figures 1A–D, 2A) and is not in line with our experiments in the SST-IRES-Cre line (Figures 5A–D). As the high number of CA3 PCL neurons in the SST-Cre mouse line was due mainly to neurons nonspecifically expressing Cre, this raises the disturbing possibility that with some viral injection protocols, even the SST-IRES-Cre mouse may display substantial nonspecific expression patterns. In line with our findings, Eyre and Bartos (2019) did not find evidence for functional direct interhemispheric inhibitory connections in the hippocampal dentate gyrus.

### The Utility of Mouse Lines With Nonspecific Principal Cell Expression for *in vivo* Experiments

A common use of Cre lines is circuit perturbation during behavioral tasks. Principal cell connections can span wide areas of the brain and must be accounted for when studying interneurons. When light is delivered to the brain through light fibers, it can travel considerable distances. Therefore, light delivered to areas where transgene expression is specific, could affect nonspecifically expressing cells and fibers in faraway areas. Notably, such effects cannot be excluded with a commonly used control group expressing only GFP (or another fluorophore) instead of a light-sensitive opsin. The same applies to a larger extent to chemogenetic experiments, where the agonist might be delivered systemically, rather than locally.

To ensure that principal cell expression does not confound a behavioral experiment, the colocalization between transgene expressing cells and the appropriate interneuron marker should be quantified for all areas where viral transduction occurred. This includes the injection cannula tract. When the transgene is expressed by crossing mouse lines, the expressing fiber distribution throughout the entire brain should be examined carefully. Especially for optogenetic experiments, it would be valuable to additionally check for direct excitatory synaptic transmission. For a specific mouse line, no direct excitatory currents should be detectable. Importantly, the net effect of a direct excitatory connection can be reduced spiking through recruitment of feedforward and feedback inhibition (Buzsáki and Czéh, 1981). Therefore, it is not sufficient to quantify spiking or activity levels in the post-synaptic population to exclude direct excitation. These issues should be considered when using any Cre-mouse line for *in vivo* behavioral experiments, particularly the SST-Cre mouse lines used in the present study.

## Data Availability Statement

Datasets are available on request. The raw data supporting the conclusions of this article will be made available by the authors, without undue reservation, to any qualified researcher.

## Ethics Statement

The animal study was reviewed and approved by Landesamt für Natur, Umwelt und Verbraucherschutz Nordrhein Westfalen.

## Author Contributions

DM-K and HB designed the project and wrote the manuscript. DM-K performed viral injections, SST antibody stainings, patch-clamp recordings and quantification of Allen Brain Institute Data. TO performed viral injections for miniSOG and performed photooxidation. MS acquired EM images and quantified them. SE performed CT-B injections and SST antibody stainings in connection with them. EA performed SST-IRES-Cre experiments and wild type viral injections.

## Conflict of Interest

The authors declare that the research was conducted in the absence of any commercial or financial relationships that could be construed as a potential conflict of interest.
